# A memory of longevity

**DOI:** 10.7554/eLife.54296

**Published:** 2020-01-24

**Authors:** Felicity Emerson, Cheng-Lin Li, Siu Sylvia Lee

**Affiliations:** 1 Biomedical and Biological Sciences ProgramCornell UniversityIthacaUnited States; 2 Department of Molecular Biology and GeneticsCornell UniversityIthacaUnited States

**Keywords:** transgenerational inheritance, epigenetics, aging, chromatin, *C. elegans*

## Abstract

Worms with increased levels of the epigenetic mark H3K9me2 have a longer lifespan that can be passed down to future generations.

**Related research article** Lee TW, David HS, Engstrom AK, Carpenter BS, Katz DJ. 2019. Repressive H3K9me2 protects lifespan against the transgenerational burden of COMPASS activity in *C. elegans*. *eLife* **8**:e48498. doi: 10.7554/eLife.48498

It is commonly accepted that genetic sequences coded within DNA are passed down through generations and can influence characteristics such as appearance, behavior and health. However, emerging evidence suggests that some traits can also be inherited ‘epigenetically’ from information that is independent of the DNA sequence.

One of the ways characteristics may be epigenetically passed down is through the temporary modification of histone proteins which help to package DNA into the cell. Histones are adorned with chemical marks that can regulate how and when a gene is expressed by changing how tightly the DNA is wrapped. These marks are typically removed before genetic information is passed on to the next generation, but some sites escape erasure (Heard and Martienssen, 2014; Kelly, 2014; Miska and Ferguson-Smith, 2016).

It has previously been reported that genetic mutations in an enzyme complex called COMPASS increase the lifespan of tiny worms called *Caenorhabditis elegans* (Greer et al., 2010). This complex acts on histones and creates a chemical mark called H3K4me, which is typically associated with less compact DNA and higher gene expression. When these mutants mate with wild-type worms they generate descendants that no longer have COMPASS mutations. Although these wild-type offspring recover normal levels of H3K4me, they still inherit the long-lived phenotype which they sustain for several generations (Greer et al., 2011). This observation suggests that an epigenetic mechanism that is independent from the gene mutation causes this inherited longevity. Now, in eLife, David Katz and co-workers at the Emory University School of Medicine in Atlanta – including Teresa Lee as first author – report a possible mechanism to explain how this longer lifespan is epigenetically inherited across multiple generations (Lee et al., 2019).

Previous work showed that one of the COMPASS complex mutants, known as *wdr-5,* has increased levels of another histone mark called H3K9me2 (Kerr et al., 2014). This epigenetic mark generally promotes DNA compaction and appears to antagonize the action of H3K4me. This led Lee et al. to question whether the elevated levels of H3K9me2 may be important for the inheritance of this extended lifespan in *wdr-5* worms.

To test their hypothesis, the team carefully monitored the levels and patterns of H3K9me2 in the mutants. Surprisingly, they found that homozygous *wdr-5* mutants, which had descended from ancestors carrying one copy of the mutated *wdr-5* gene and one wild-type copy for multiple generations, did not live for longer than their non-mutant counterparts. This indicates that the mutation carried by *wdr-5* worms did not immediately cause a lifespan change. However, future generations of worms that maintained the homozygous *wdr-5* mutation had an increasingly longer lifespan, suggesting that the accumulation of an epigenetic signal across generations promotes longer living. These late generation *wdr-5* mutants had higher levels of H3K9me2, and they were able to pass on this extended longevity to their progeny following mating with wild-type worms as previously reported (Greer et al., 2011).

Next, Lee et al. manipulated the levels of H3K9me2 to see how this affected the phenotype of the late generation, long-lived *wdr-5* worms. First, they blocked the gain in H3K9me2 levels in the mutants by introducing a defective version of an enzyme called MET-2 which normally promotes the addition of H3K9me2 (Figure 1). As a result, neither the *wdr-5* mutants nor their descendants experienced a longer lifespan. Lee et al. reasoned that if higher H3K9me2 levels are responsible for longevity, then increasing the amount of H3K9me2 by a different mutation should result in the same phenotype as the *wdr-*5 worms. They found that worms with defects in the enzyme JHDM-1, which is predicted to remove H3K9me2, not only lived longer but also passed on this trait to their wild-type progeny for several generations. Together, these data strongly suggest that increased H3K9me2 levels contribute to extended longevity and its inheritance.

**Figure 1.**
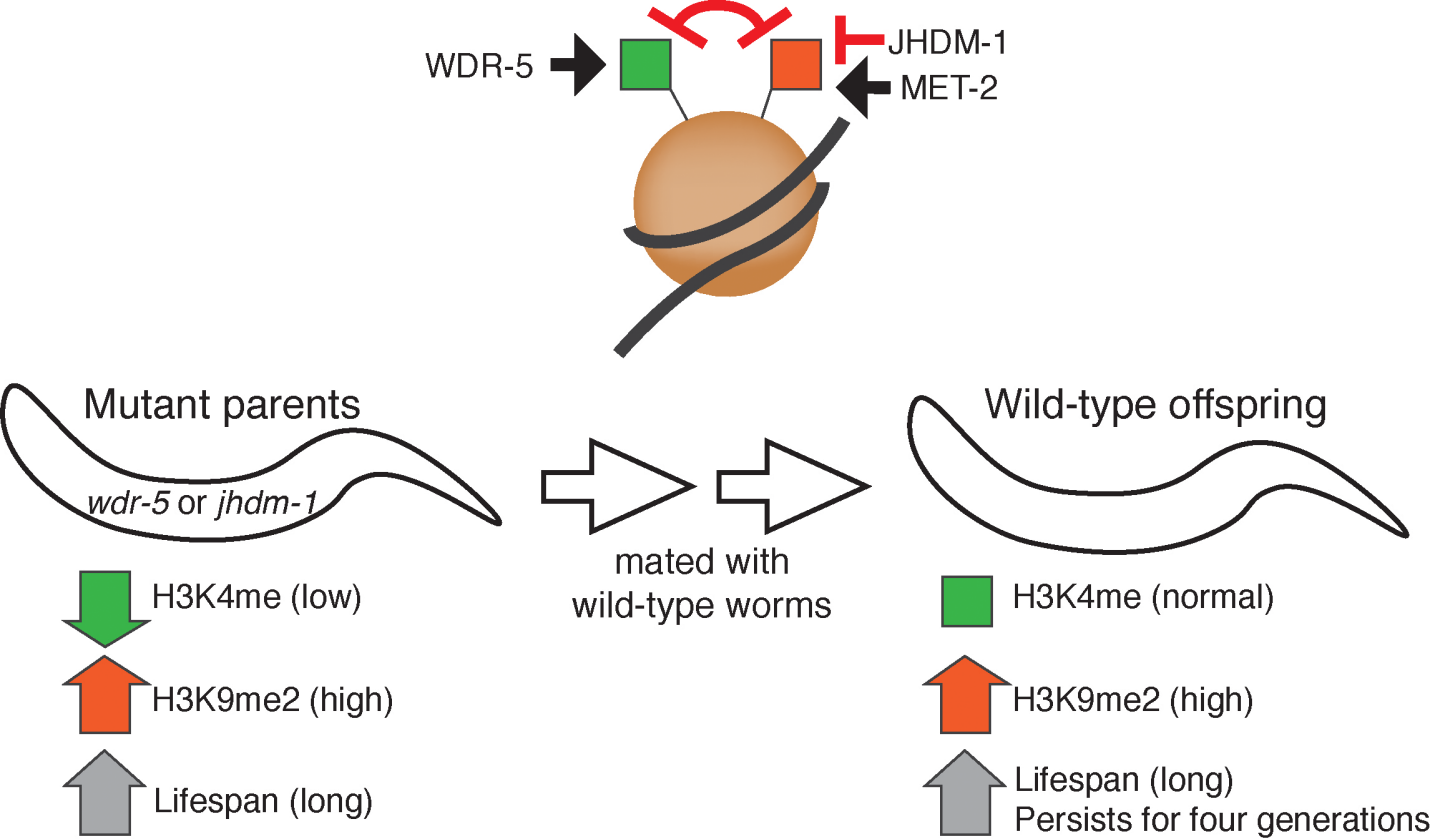
Certain epigenetic changes are linked to the inheritance of extended lifespans in worms. Top: The WDR-5 enzyme helps to place the H3K4me mark (green), which promotes gene expression, on proteins called histones (brown circle) that package DNA (grey ribbon). In parallel, the MET-2 enzyme places the H3K9me2 mark (red), which represses gene expression. The two marks functionally antagonize each other. An enzyme called JHDM-1 is predicted to remove H3K9me2. Bottom: Worms with mutations in *wdr-5* or *jhdm-1* (left) that have low levels of H3K4me (green arrow), also show higher levels of H3K9me2 (red arrow) and an increased lifespan (grey arrow). When these long-lived mutants are mated to wild-type worms with a normal lifespan, their genetically wild-type offspring (right) are still long-lived for several generations (grey arrow). These worms show normal levels of H3K4me mark (green square) and regions of sustained increase in H3K9me2 (red arrow) inherited from their mutant ancestors.

Image credit: Cheng-Lin Li.

To build on these findings, Lee et al. explored where the H3K9me2 marks were deposited in the genomes of the worms. As expected, long-lived *wdr-5* and *jhdm-1* mutants have more H3K9me2 marks spread across their genomes. Critically, Lee et al. found that specific genes in the wild-type offspring of *jhdm-1* mutants had higher levels of H3K9me2. These results are intriguing and suggest that increasing H3K9me2 levels in certain genes may be the key to passing on this long living phenotype to future generations. Exciting future investigations will be to identify all the gene regions associated with the inherited increase in H3K9me2, and to understand how changes to DNA packaging and gene expression in those regions influence longevity.

A handful of previous studies in *C. elegans* have demonstrated that specific histone modifications can be inherited across generations (Katz et al., 2009; Kerr et al., 2014; Kaneshiro et al., 2019). However, the paper by Lee et al. is the first to tie together the inheritance of a histone mark to longer lifespan. H3K9me2 is an evolutionarily conserved histone mark which is known to preserve spatial organization during cell division in organisms ranging from humans to worms (Poleshko et al., 2019). Going forward, it will be interesting to study whether H3K9me2 also participates in how traits are inherited across multiple generations in mammals.
